# Liver Resection for Intrahepatic Cholangiocarcinoma—Single-Center Experience with 286 Patients Undergoing Surgical Exploration over a Thirteen Year Period

**DOI:** 10.3390/jcm10163559

**Published:** 2021-08-13

**Authors:** Hauke Lang, Janine Baumgart, Stefan Heinrich, Tobias Huber, Lisa-Katharina Heuft, Rabea Margies, Jens Mittler, Felix Hahn, Tiemo S. Gerber, Friedrich Foerster, Arndt Weinmann, Jens U. Marquardt, Roman Kloeckner, Beate K. Straub, Fabian Bartsch

**Affiliations:** 1 Department of General, Visceral and Transplant Surgery, University Medical Center of the Johannes Gutenberg-University Mainz, 55131 Mainz, Germany; janine.baumgart@unimedizin-mainz.de (J.B.); stefan.heinrich@unimedizin-mainz.de (S.H.); tobias.huber@unimedizin-mainz.de (T.H.); lisa-katharina.heuft@unimedizin-mainz.de (L.-K.H.); rabea.margies@unimedizin-mainz.de (R.M.); jens.mittler@unimedizin-mainz.de (J.M.); fabian.bartsch@unimedizin-mainz.de (F.B.); 2 Department of Diagnostic and Interventional Radiology, University Medical Center of the Johannes Gutenberg-University Mainz, 55131 Mainz, Germany; felix.hahn@unimedizin-mainz.de (F.H.); roman.kloeckner@unimedizin-mainz.de (R.K.); 3 Department of Pathology, University Medical Center of the Johannes Gutenberg-University Mainz, 55131 Mainz, Germany; tiemo.gerber@unimedizin-mainz.de (T.S.G.); beate.straub@unimedizin-mainz.de (B.K.S.); 4 1st Department of Internal Medicine, Gastroenterology and Hepatology, University Medical Center of the Johannes Gutenberg-University Mainz, 55131 Mainz, Germany; friedrich.foerster@unimedizin-mainz.de (F.F.); arndt.weinmann@unimedizin-mainz.de (A.W.); 5 1st Department of Medicine, University Medical Centre Schleswig-Holstein, Campus Lübeck, 23538 Lübeck, Germany; jens.marquardt@uksh.de

**Keywords:** intrahepatic cholangiocarcinoma, cholangiocarcinoma, survival, liver resection, repeated liver resection

## Abstract

Background: Intrahepatic cholangiocarcinoma (iCCA) accounts for about 10% of primary liver cancer. Surgery is the only potentially curative treatment. We report on our current series of 229 consecutive hepatic resections for iCCA, which is one of the largest Western single-center series published so far. Methods: Between January 2008 to December 2020, a total of 286 patients underwent 307 surgical explorations for intended liver resection of iCCA at our department. Data were analyzed with regard to (1) preoperative treatment of tumor, (2) operative details, (3) perioperative morbidity and mortality, (4) histopathology, (5) outcome measured by tumor recurrence, treatment of recurrence and survival and (6) prognostic factors for overall and disease-free survival. Results: the resectability rate was 74.6% (229/307). In total, 202 primary liver resections, 21 repeated, 5 re-repeated, and 1 re-re-repeated liver resections were performed. In primary liver resections there were 77% (155/202) major hepatectomies. In 39/202 (20%) of patients additional hepatic wedge resections and in 87/202 (43%) patients additional 119 other surgical procedures were performed next to hepatectomy. Surgical radicality in first liver resections was 166 R0-, 33 R1- and 1 R2-resection. Following the first liver resection, the calculated 1-, 3- and 5-year-survival is 80%, 39%, and 22% with a median survival of 25.8 months. Until the completion of data acquisition, tumors recurred in 123/202 (60.9%) patients after a median of 7.5 months (range 1–87.2 months) after resection. A multivariate cox regression revealed tumor size (*p* < 0.001), T stage (*p* < 0.001) and N stage (*p* = 0.003) as independent predictors for overall survival. N stage (*p* = 0.040), preoperative therapy (*p* = 0.005), T stage (*p* = 0.004), tumor size (*p* = 0.002) and M stage (*p* = 0.001) were independent predictors for recurrence-free survival. Conclusions: For complete surgical removal, often extended liver resection in combination with complex vascular or biliary reconstruction is required. However, despite aggressive surgery, tumor recurrence is frequent and long-term oncological results are poor. This indicated that surgery alone is unlikely to make great strides in improving prognosis of patients with iCCA, instead clearly suggesting that liver resection should be incorporated in multimodal treatment concepts.

## 1. Introduction

Intrahepatic cholangiocarcinoma (iCCA), although less frequent than perihilar cholangiocarcinoma (pCCA) is the second most common primary liver tumor after hepatocellular carcinoma (HCC). It accounts for about 10% of primary liver malignancies but shows an increasing incidence in Western countries within the past decade [1]. Due to its intrahepatic and often peripheral localization, tumor related symptoms usually occur late in the course of the disease. Therefore, the majority of tumors are diagnosed in an already locally advanced or even metastatic stage when curative approaches are difficult. In general, the prognosis of patients with iCCA is poor with a reported median survival of about one year after diagnosis and a 5-year survival of about 10% only [2]. Liver resection is the standard of care if a potentially curative approach is intended [3,4,5,6,7]. However, due to the rarity of iCCA, data on liver resection are still limited as most series evaluating surgical therapy of iCCA are based on very small patient cohorts. In addition, in many series there is no clear differentiation between intra- and extra-hepatic cholangiocarcinoma, and outcome analysis is hampered by the fact that data have been collected over time periods exceeding one or even two decades. Due to ongoing progress in diagnostics, prognostication, and advances in liver surgery techniques as well as new multimodal treatment options, comparability of data is very limited.

Since 2008 we have adopted an aggressive surgical attitude in the treatment of iCCA. This study analyzes our temporary series of 229 consecutive resections for iCCA within the past thirteen years which is to the best of our knowledge one of the largest Western single-center series in the literature.

## 2. Materials and Methods

All patients undergoing surgical exploration and liver resections at our center are registered in a prospective institutional database. Patients who underwent liver surgery for intrahepatic cholangiocarcinoma (iCCA) from 2008 to 2020 were eligible for this analysis.

The diagnosis of iCCA was based on histology obtained either by pre- or intra-operative biopsy or by the resected specimen. Patients with hilar cholangiocarcinoma, gallbladder carcinoma, mixed hepatocellular/cholangiocarcinoma, or bile duct carcinoma not clearly attributable to the intrahepatic biliary tree as well as patients with severe parenchymal damage (cirrhosis, fibrosis > F2 or steatosis > 50%) were excluded from our analysis.

**Figure 1:**
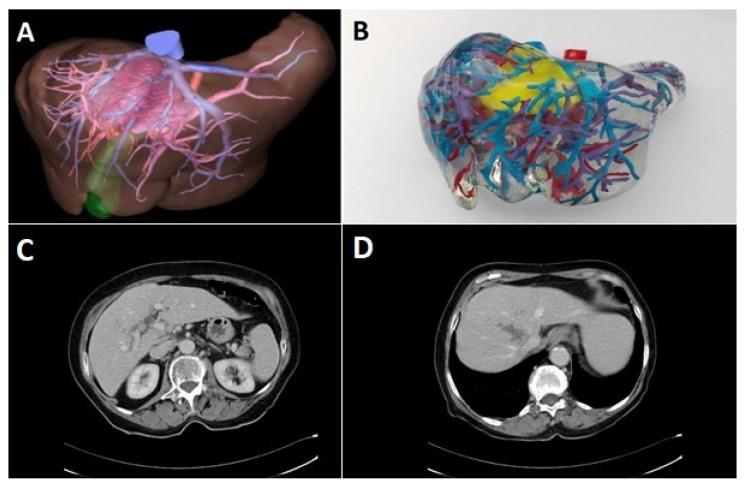
Preoperative 3D reconstruction as a PDF presentation (**A**) and 3D-print (**B**) with stained polyurethane rubber of the liver as well as CT-scan (**C**,**D**) in a 70-year-old patient with iCCA that infiltrated the right and middle hepatic vein and had contact to the left hepatic vein. Preoperative volumetric analysis of the segments 2/3 revealed a remnant volume of 563 mL. Resection was performed as an extended right-sided hemihepatectomy with hilar resection and reconstruction of the left portal vein and the medial of two branches of the left hepatic vein.

Preoperative diagnostic work-up included ultrasound of the abdomen and computed tomography (CT) of the abdomen and chest. Upper and lower gastrointestinal endoscopy was performed to exclude extrahepatic primary tumor in cases where the diagnosis of an iCCA was not made by biopsy. In selected cases, three-dimensional CT-scan of the liver including volumetry, virtual tumor resection, and computer-assisted risk analysis was performed prior to the resection. These were performed either by an external provider (MeVis Distant Services, MeVis AG, Bremen, Germany) or with a local reconstruction software (Synapse 3D, Fujifilm AG, Tokyo, Japan) by a trained surgical resident [8]. Since 2017, 3D-prints of the liver were performed on special request of the surgeon with non-flexible polyurethane rubber [9]. Especially in cases with anticipated complex vascular reconstructions, 3D-prints were ordered for preoperative planning (Figure 1).

All surgical explorations and resections were performed by a team of experienced surgeons with special expertise in hepato-biliary surgery, and retrospectively classified according to the “New World” terminology [10]. Postoperatively, for at least two years, we conducted follow-up every three months; later on, the interval was increased to 6 months, if reasonable. Preferably, CT imaging was obtained at least every 6 months alternating with ultrasound examinations. For patients who were not able to undergo follow-up at our center, further information was retrieved from treating physicians.

The data collection was completed in February 2021. The data of the patients undergoing liver resection (*n* = 223) were further analyzed with regard to (1) preoperative treatment of tumor, (2) operative details, (3) perioperative morbidity and mortality, (4) pathologic findings, (5) outcome measured by tumor recurrence, treatment of recurrence and survival, and (6) prognostic factors for overall and disease-free survival. Surgical complications were assessed according to the Dindo–Clavien classification [11]. The TNM classification was performed according to the 8th edition of the classification of the Union for International Cancer Control (UICC) [12] (Table 1).

All patients signed an informed consent that allowed the data and follow-up to be collected anonymously and potentially used for scientific analysis. Abiding by the regulations of the federal state law (state hospital law §36 & §37) and according to the independent ethics committee of Rhineland-Palatinate, no ethical approval was necessary for this study.

### Statistical Analyses

SPSS 23 (IBM Corp. Released 2015. IBM SPSS Statistics for Windows, Version 23.0. Armonk, NY, USA: IBM Corp.) was used to perform statistics. Categorical data was analyzed using the Chi2 test in cross tabulation. Survival analyses were conducted with the Kaplan Meier model and for comparison of factors influencing survival the log rank test was utilized. A *p*-value of <0.05 was considered significant. All analyses were an intention to treat, and no patients were excluded. Recurrence-free survival was defined according to Punt and colleagues [13].

## 3. Results

During the study period from January 2008 to December 2020, a total of 286 patients (137 female and 149 male) with a median age of 65 years (range: 28–84 years) underwent 307 surgical explorations for intended liver resection of iCCA at our department. This included 33 explorations for recurrent iCCA (parts of these data were already published in [14,15,16,17]).

### 3.1. Surgical Procedures and Intraoperative Data

**Figure 2:**
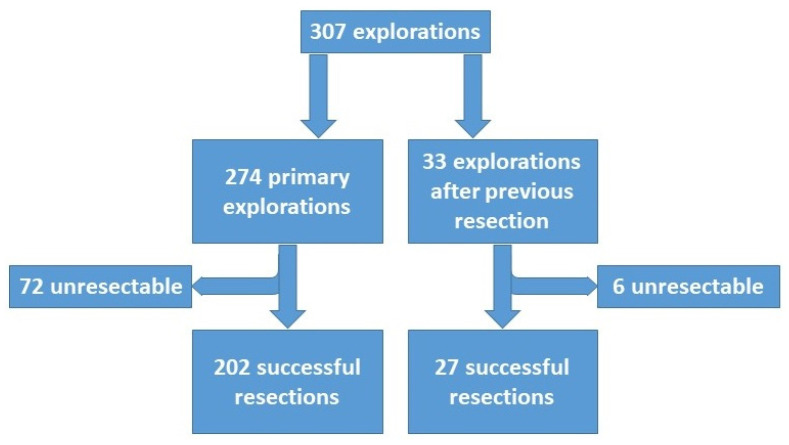
Flowchart of all explorations. Further subdivision in primary and repeated explorations.

**Figure 3:**
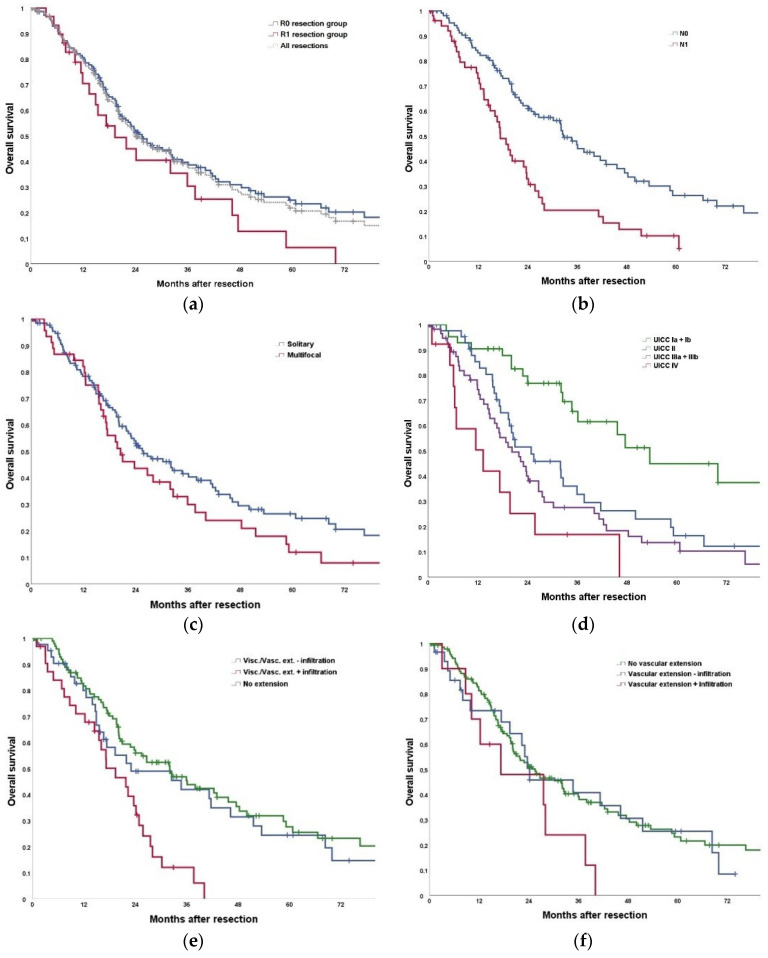
(**a**) Comparison of overall survival of patients with R0 versus R1 resection; *p* = 0.092. Additionally, combined depiction of the complete cohort. Perioperative deaths were excluded. (**b**) Comparison of overall survival of patients with N0 or N1 status; *p* < 0.001. Perioperative deaths were excluded. (**c**) Comparison of overall survival of patients with solitary versus multifocal iCCA; *p* = 0.144. Perioperative deaths were excluded. (**d**) Comparison of overall survival of different UICC groups; *p* < 0.001. Subgroup comparison: UICC I vs. UICC II *p* = 0.002; UICC I vs. UICC III *p* < 0.001; UICC I vs. UICC IV *p* < 0.001; UICC II vs. UICC III *p* = 0.252; UICC II vs. UICC IV *p* = 0.014; UICC III vs. UICC IV *p* = 101. Perioperative deaths and patients with Nx status were excluded. (**e**) Comparison of overall survival of patients with visceral and/or vascular extension (VVE) versus no extension; *p* < 0.001. Subgroup comparison: no extension vs. VVE − infiltration *p* = 0.465; no extension vs. VVE + infiltration *p* < 0.001; VVE − infiltration vs. VVE + infiltration *p* = 0.007. Perioperative deaths were excluded. (**f**) Comparison of overall survival of patients without vascular extension (VE), VE without (−) infiltration and VE with (+) infiltration; *p* = 0.163. Subgroup comparison: no VE vs. VE − infiltration *p* = 0.746; no VE vs. VE + infiltration *p* = 0.058; VE − infiltration vs. VE + infiltration *p* = 0.125. Perioperative deaths were excluded.

The overall resectability rate was 74.6% (229/307) for all explorations, 73.7% (202/274) in attempted first liver resection and 81.8% (27/33) in repeated resection (Figure 2). In total, 202 primary liver resections, 21 repeated, 5 re-repeated and 1 re-re-repeated liver resection were performed (Table 2). In primary liver resections, there were 77% (155/202) major hepatectomies (resection of three or more segments). In addition, the caudate lobe was removed in 53 patients, and in another 39 patients, additional atypical resections were performed to remove satellite lesions. In 87/202 (43%) patients, 119 additional surgical procedures were performed in addition to hepatectomy (Table 3; Figure 3e,f).

### 3.2. Preoperative Treatment

Preoperatively, endoscopic intraductal stents or percutaneous transhepatic cholangiodrainages to relieve jaundice had been placed in 10 and 3 patients, respectively. One patient underwent both procedures. Prior to the first liver resection, two patients had undergone loco-regional therapy for iCCA with chemoembolization (*n* = 1) or radiofrequency ablation (*n* = 1). In 18 patients with large iCCA considered to be irresectable, secondary resectability was achieved after chemotherapy and downsizing of tumors. Prior to repeated liver resection (*n* = 21), one patient was treated with chemotherapy. Thirteen patients had undergone their first liver resection in our department and eight patients in referring hospitals.

### 3.3. Morbidity and Mortality

After primary resection, a total of 131 complications (Clavien–Dindo grade I–IV) occurred in 80 of 202 patients (39.6%). The distribution of complications (highest complication only) is listed in Table 4. The overall 90-day-mortality rate was 7.9%.

### 3.4. Postoperative Treatment

A total of 60 patients underwent adjuvant chemotherapy, most often with capecitabin (*n* = 44) followed by gemcitabine and cisplatin (*n* = 8). Another eight patients were included in the ACTICCA trial [18]. Two patients underwent postoperative radiation therapy.

### 3.5. Pathology

The median tumor diameter (in case of multifocal tumor the diameter of the largest nodule) was 7 cm (range 4–20 cm). Tumors were solitary in 150 patients (74.3%; Figure 3c). Overall, in the first liver resections, there were 166 R0 resections, 33 R1 resections and 1 R2 resection. In two cases, resection status was not determined (Rx) (Table 5; Figure 3a). Repeated resection resulted in 24 R0 resections and three R1 resections.

Vascular infiltration was present in 16/202 patients and in 16/55 resected vessels, respectively. Infiltration of the vena cava and portal vein was found in 6/16 cases (37.5%) each, whereas there was infiltration of the vascular wall of only 4/22 (18%) of resected major liver veins. The only resected and reconstructed artery did not show tumor invasion (Table 3).

At primary liver resection, lymphadenectomy was performed in 89.6% (181/202) of cases. A median number of 5 lymph nodes was harvested (range 0–31). Lymph node metastasis were seen in 58/181 (32%) patients (Table 5; Figure 3b). In the repeated liver resection, no more lymphadenectomy was performed.

### 3.6. Survival, Recurrence, and Treatment of Recurrence

Following the first liver resection, the calculated 1-, 3- and 5-year-survival is 80%, 39%, and 22% with a median survival of 25.8 months (Figure 3a). Until the completion of data collection for this analysis, tumor recurred in 60.9% (123/202) of patients after a median of 7.5 months (range 1–87.2 months) after resection. The underlying tumor stages were UICC Ia (*n* = 6/22), Ib (*n* = 16/32), II (*n* = 36/47), IIIa (*n* = 4/9), IIIb (*n* = 40/58) and IV (*n* = 9/13); due to Nx UICC stage was not assessable in 12/21 patients (Figure 3d). The initial site of recurrence was intrahepatic only in 54 (44%), extrahepatic only in 28 (23%) and both intra- and extra-hepatic in 41 (33%) patients. Treatment of recurrence consisted of repeated hepatectomy (*n* = 13), resection of extrahepatic tumor (*n* = 1), chemotherapy (*n* = 78) and/or chemoembolization (*n* = 6), local ablation (*n* = 7), radiation/chemoradiation (*n* = 3) or best supportive (*n* = 15) only.

So far, 100 of the 123 patients with recurrent tumor have died after a median time of 19.9 months (range: 3.1–104.7 months) after resection and 12.8 months (range 0.5–54.7 months) after diagnosis of recurrence. A total of 23 patients (18.7%) are alive with a median survival of 29 months (range 7.6–130.4 months) after initial resection and 20.7 months (range 1–115.3 months) after diagnosis of recurrence.

### 3.7. Outcome after Associating Liver Partition and Portal Vein Ligation (ALPPS)

In total, eight ALPPS were performed for solitary (*n* = 3) and multifocal (*n* = 5) iCCA. There were eight R0 resections. In multifocal iCCA, all patients died within 22 months after liver resection. So far, the three patients with ALPPS for solitary iCCA are alive without evidence of recurrence 11.5 years, 3 years, and 5 months after ALPPS. The patient being alive 11.5 years after ALPPS had recurrent iCCA one year after initial resection. This patient underwent repeated resection (wedge resection) and is now alive without evidence of repeated recurrence 10.5 years after repeated resection and 11.5 years after ALPPS, respectively.

### 3.8. Exploration Group

Palliative chemotherapy was performed in 62 patients with various protocols most of them based on Gemcitabine and Cisplatin. Further treatments included transarterial chemoembolization (TACE, *n* = 3), selective internal radiotherapy (SIRT, *n* = 2), radiation (*n* = 2) or best supportive care (*n* = 4). So far, 45/62 patients have died after a median of 9.6 months (range: 0.6–59.8; IQR: 3.9–20.9), 17 are alive at a follow-up between 6 and 59.8 months.

### 3.9. Predictors of Survival and Recurrence-Free Survival

In univariate analysis the following factors had significant influence on overall survival: extended resection, visceral extension, vascular infiltration, visceral infiltration, tumor size, T-stage, N-stage, M-stage, Pn-stage, and UICC stage (Table 6). In a multivariate cox regression tumor size (*p* < 0.001), T stage (*p* < 0.001) and N stage (*p* = 0.003) were predictors for overall survival (Table 7).

For recurrence-free survival upon univariate analysis the following parameters had significant influence: extended resection, visceral extension, tumor size, multifocality, T-stage, N-stage, M-stage and UICC stage (Table 6). In multivariate cox regression analysis N stage (*p* = 0.040), preoperative therapy (*p* = 0.005), T stage (*p* = 0.004), tumor size (*p* = 0.002) and M stage (*p* = 0.001) were independent predictors for recurrence-free survival (Table 7).

## 4. Discussion

Surgical treatment of iCCA is still one of the main challenges in hepatobiliary surgery. Most often, iCCAs are diagnosed late in the course of the disease when tumors are locally advanced or even in a metastatic stage. In our series, the vast majority of tumors required major hepatectomy (in almost 80%) and additional operative procedures such as complex vascular and biliary reconstructions in nearly 50% of our cases, exceeding in this regard other reports by far. Thus, the presented series is one of the largest in the Western world, not just because of the numbers but also with regard to complexity of procedures. Further, as a single center series pursuing the same aggressive surgical strategy over the entire inclusion period, the comparability of data is also relatively well in contrast to often inhomogeneous multicentric data.

The reported survival after hepatectomy for iCCA ranges between 31% and 59% at 3 years and 21% to 45% at 5 years, depending on the selection criteria for surgery (Table 8). The herein presented results with a 3- and 5-year-survival of 35% and 22% are at the lower range of the reported data, but in our series the extent of liver resection and additional procedures such as vascular reconstructions exceeded those of most other reports by far, indicating more advanced tumors and more difficult resections [19,20]. Looking exclusively at the subgroup undergoing hepatectomy without operative extension, the results with 46% and 28% survival at 3- and 5-years are in accordance with most published data. These results are comparable to the results of surgical therapy in many other gastrointestinal malignancies, endorsing that therapeutic nihilism is not justified in iCCA. However, the presented data also clearly indicate that even with an aggressive surgical approach the chance of cure is still small in iCCA.

In our series, there was resection/reconstruction of major hepatic vessels or the inferior vena cava in 22% of the cases. Notably, pathology confirmed tumor infiltration of major vessels in only 29% of the suspected cases. As infiltration is difficult to assess by preoperative or intraoperative imaging, our approach is to resect and reconstruct vessels whenever infiltration is suspected. This aggressive approach seems to be justified as this can be done with acceptable low morbidity and mortality.

The most aggressive surgical approach to iCCA is ALPPS [35]. Our results after ALPPS for iCCA are consistent with data from a multicentric analysis by Li et al. [36]. While there seems to be hardly any benefit of such an aggressive approach in multifocal tumors ALPPS seems to be justified in selected cases of solitary iCCA. The patient surviving 11.5 years is to the best of our knowledge the longest survivor worldwide after an ALPPS procedure at all.

Depending on the aggressiveness of the surgical approach and the quality of preoperative diagnostic and staging procedures reported resectability rates of iCCA show great variability ranging between 50% and 75% [14,23,37,38]. This is considerably lower than in most if not in all other hepatobiliary malignancies. Most often multinodular intrahepatic tumor spread or less often peritoneal seedings are the main causes of irresectability. In our series the resectability rate of almost 80% is high but this is at the expense of the need for often extensive resections with frequently complex resections and reconstruction of adjacent structures. We follow this aggressive approach as resection offers the only chance for cure.

It has already been shown that routine use of staging laparoscopy results in a reduction of about 20% of explorative laparotomies in biliary cancer [39,40]. Avoiding a large laparotomy can help to guide these patients towards an immediate palliative treatment. In addition, computer-assisted operation planning and 3D reconstruction of liver anatomy that may provide significant anatomical information. In recent years, we used 3D-prints of the liver for operation planning in selected cases [9]. Although very instructive, it needs to be evaluated whether these techniques can also contribute to a better assessment of resectability, to a reduction of perioperative morbidity and mortality and finally to an improvement of the oncological outcome.

One aim of our analysis was to identify prognostic markers and risk factors for tumor recurrence after hepatic resection. Like other studies, we could confirm extended resection, visceral extension, vascular infiltration, visceral infiltration, tumor size, T-stage, N-stage, M-stage, Pn-stage, and UICC stage to be significantly associated with tumor recurrence and poor survival after R0 resection. In multivariate analysis, tumor size, T stage and N stage were significantly associated with a worse overall survival, while N stage, preoperative therapy, T stage, tumor size and M stage were associated with worse recurrence-free survival. Therefore, based on the presented data, we do not consider the presence of any of these prognostic factors (with the exception of UICC stage IV) as a contraindication to liver resection. In particular, we do not consider the potential need for extension of resections a contraindication, the more as even modern imaging modalities do not reliably predict macro- or micro-vascular infiltration.

There is agreement that resection of stage IV iCCA should only be performed in selected cases in palliative intention where tumor associated symptoms cannot be controlled otherwise. However, there is still discussion about surgical therapy in the presence of lymph node involvement [41]. Up to now, there is no evidence whether lymphadenectomy is of prognostic value only or also beneficial for survival [42]. Our data with a median survival of 18 months and a 5-year-survival of 12% after R0 resections in the presence of lymph node metastases suggest a probable survival benefit of liver resection. However, in this patient group the need for effective perioperative therapy is highly evident.

The new UICC classification recommends the removal of at least 6 lymph nodes from the hilar region and along the lesser gastric curve in left-sided iCCA or retropancreatic in right-sided iCCA [12]. Hopefully, this will help to further improve both treatment stratification of iCCA and, with ongoing progress in adjuvant chemotherapy, also oncological outcome.

Tumor recurrence is by far the most frequent cause of death after resection of iCCA [43,44,45]. Our data reveal that the liver is the most frequent primary site of tumor recurrence with 50% of cases where the liver is the only initial site of recurrence. We performed repeated resection in 27 cases with a survival even longer than in the entire resection group [16]. In a recent German multicenter study with 113 repeated resections, mainly by minor hepatectomies or segmentectomies, a 3- and 5-year disease-free survival of 36% and 28% was reported [46]. These survival rates are even slightly better than after primary resection, suggesting that there might be a selection bias indicating a more favorable biology in those recurrent tumors remaining confined to the liver [47].

Due to the often-advanced disease at the time of diagnosis, there is a substantial number of cases where an R1 resection cannot be avoided. In particular, in large or centrally located tumors, sometimes there is no more margin to be left or final histology shows microscopic tumor invasion of the resection margin which at operation had been assumed to be tumor-free. Similarly, although in the current series all resections were performed with curative intention, we achieved an R1-resection rate of 16%. In former years, R1 resection was supposed to provide little survival benefit rather than to face the patients to the risks of major hepatic surgery [48,49,50,51]. However, with the availability of effective chemotherapy, the value of R1 resections probably needs to be re-evaluated, in particular in combination with neoadjuvant and downsizing therapy strategies [52].

Although significantly better than the data of non-resected tumors, the achieved long-term results even after extensive resections with complex reconstructions are poor. This indicates that there is a limit to the contribution made by radical resections alone. Hence, since the first results of the BILCAP trial (adjuvant capecitabine versus observation following R0/R1 resection of bile duct cancer) became available, adjuvant chemotherapy with capecitabine is currently the standard of care after resection of iCCA in our institution [53].

In our series, we had 18 resections following neoadjuvant CTx for down-sizing. The survival data in these patients are at least comparable with those of patients undergoing upfront surgery without chemotherapy. Assuming that the tumors in the downsizing group were more advanced, these results suggest the effectiveness of CTx. Consistently, in a multivariate analysis, preoperative chemotherapy was one prognostic factor significantly associated with improved recurrence-free survival. Certainly, our series is too small to draw any valid conclusion, but our data are in line with some recent reports from the literature. In a French study neoadjuvant chemotherapy led to a secondary resectability in 39/74 (53%) patients with initially borderline resectable or irresectable iCCA [54]. These patients had survival rates similar to the group with initially resectable iCCA. Similarly, in a recent propensity-matched analysis (203 versus 487 patients with iCCA) based on data from the National Cancer Database (NCDB, USA), neoadjuvant treatment was associated with a significantly higher R0 resection rate and better survival compared to adjuvant therapy (median OS: 40.3 vs 32.8 months; *p* = 0.01) [55]. This suggests the potential effectiveness of neoadjuvant (downsizing) treatment for iCCA and justifies further evaluation of this concept [56,57].

Further, cholangiocarcinoma has become a hallmark of modern precision medicine. With the development of next-generation sequencing (NGS) targeted therapies become increasingly available. In the palliative setting, first promising results have been reported after treatment with fibroblast growth factor receptor (FGFR) and isocitrate dehydrogenase (IDH) inhibitors [58]. In future it is possible that these targeted therapies may also play a role in preoperative neoadjuvant or downsizing therapy [59,60].

## 5. Conclusions

In conclusion, our results suggest that complete surgical resection may provide prolonged survival even in locally advanced but not metastatic iCCA. As iCCA is usually diagnosed late in the course of disease, often extended liver resection in combination with complex vascular or biliary reconstruction is necessary to achieve complete tumor removal. Improved surgical techniques, in particular those derived from liver transplant surgery, have continuously pushed the frontiers of liver surgery. However, as is the case for iCCA, the limits of what is technically feasible are often reached. Nevertheless, even when pulling out all stops of surgical skills, the long-term oncological results have remained poor, indicating that surgery alone is unlikely to make significant strides in improving the prognosis of iCCA. This strongly suggests that surgery of iCCA should be integrated into multimodal perioperative treatment concepts. Recent results have shown the efficacy of adjuvant chemotherapy, and there are also some promising results with neoadjuvant therapy, in particular regarding downsizing therapy to achieve secondary resectability. Further studies are needed to better evaluate treatment options in order to shed further light on this topic.

## Figures and Tables

**Table 1:** Definitions of the 8th edition of the UICC classification.

T-Stage		N-Stage		UICC-Stage	
T1a	Solitary tumor ≤5 cm without vascular invasion	N0	No regional lymph node metastases	UICC Ia	T1a N0 M0
T1b	Solitary tumor ≥5 cm without vascular invasion			UICC Ib	T1b N0 M0
T2	Solitary tumor with intrahepatic vascular invasion or multiple tumors, with or without vascular invasion	N1	Regional lymph node metastasis present	UICC II	T2 N0 M0
T3	Tumor perforating the visceral peritoneum	Recommendation of harvesting of at least 6 lymph nodes		UICC IIIa	T3 N0 M0
T4	Tumor with infiltration of local extrahepatic structures			UICC IIIb	T4 and/or N1, M0
				UICC IV	any T, any N, M1

**Table 2:** Operative Procedures in liver resections.

**Operative Procedures in 202 Primary Liver Resections**			
**Primary Surgery**		***n* = 202**	**%**
Bisegmentectomy		31	15.3
Monosegmentectomy		14	6.9
Subsegmentectomy		2	1
Resection of three liver segments		13	6.4
Right hemihepatectomy		27	13.4
Left hemihepatectomy		31	15.3
Mesohepatectomy (≥three central segments)		11	5.4
Right trisectionectomy		33	16.3
Left trisectionectomy		32	15.8
ALPPS		8	4
Additional liver resections *		in 86 patients	
Caudate lobectomy		53	
Wedge resection		39	
**Operative Procedures in 27 Repeated Liver Resections**			
**Repeated Resections**	**1st Rep. Expl.**	**2nd Rep. Expl.**	**3rd Rep. Expl.**
	***n* = 21**	***n* = 5**	***n* = 1**
Hemihepatectomy	1	-	-
Bisegmentectomy	6	1	-
Monosegmentectomy	6	3	-
Subsegmentectomy	7	1	1
Extrahepatic lymph node resection	1	-	-
Repeated exploration	6	-	-

* Procedures performed in addition to the main surgical procedure. rep. expl. = repeated exploration.

**Table 3:** Extensions of primary resection (*n* (%)).

**Visc/Vasc Extension**	**87 (100)**	
Visceral only	43 (49.5)	
Vascular only	27 (31)	
Both	17 (19.5)	
**Vascular Extension**	Cases *n* = 44 *	**Infiltration *n* (%)**
Hepatic artery	1	0 (-)
Portal vein	16	6 (37.5)
Major hepatic vein	22	4(18.2)
Vena cava inferior	16	6 (37.5)
**Visceral Extension**	Cases *n* = 60 *	**Infiltration *n* (%)**
Diaphragm	12	4 (33.3)
Adrenal gland	5	2 (40)
Hilar bifurcation	43	19 (44.2)
Pericardium	1	1 (100)
Duodenum	1	1 (100)
Colon	1	1 (100)
Stomach	1	1 (100)

* Cases are the number of patients in which visceral or vascular extensions were performed. In some patients more than one extension was performed therefore the total number of extensions is unlike the number of cases.

**Table 4:** Morbidity after primary resection.

Morbidity	Primary Resection *n* = 202
Bile leakage	42
Intraabdominal abscess	20
Portal vein thrombosis	8
Massive pleural effusion	5
Massive ascites	10
Wound infection	17
Pleural empyema	1
Ileus	3
Bile duct stenosis	1
Pneumonia	11
Bleeding	9
Cardial event	5
Highest Dindo–Clavien cl.	
No morbidity	104
Type I	8
Type II	18
Type IIIa	35
Type IIIb	8
Type IVa	7
Type IVb	4

cl. = classification.

**Table 5:** Histopathological staging after primary liver resection.

	*n* = 202
Solitary tumors	150
Multifocal tumors	52
Tumor size in cm * (median (range))	7 (4–20)
Lymph nodes harvested (median (range))	5 (0–31)
**R-stage**	
R0	166
R1	33
R2	1
Rx	2
**T-stage**	
T1a	34
T1b	51
T2	76
T3	15
T4	26
**N-stage**	
N0	123
N1	58
Nx	21
**L-stage**	
L0	168
L1	34
**V-stage**	
V0	155
V1	43
V2	4
**Pn-stage**	
Pn0	144
Pn1	58
**Grading**	
G1	3
G2	129
G3	51
G4	1
No grading **	18
**Clinical M-stage**	
cM0	188
M1	14
**UICC-stage ‡**	
Ia	23
Ib	31
II	47
IIIa	9
IIIb	58
IV	13

* Size of largest nodule, if multifocal; ** after application of preoperative chemotherapy; ‡ due to Nx status 21/202 patients were not classified.

**Table 6:** Univariate survival analysis.

	Kaplan Meier	
	OS	RFS
Age	0.329	0.334
Gender	0.336	0.097
ASA classification	0.723	0.317
Preoperative biopsy	0.388	0.515
Preoperative therapy	0.886	0.053
Major resection	0.072	0.065
Extended resection	**0.040**	**0.039**
Vascular extension	0.290	0.326
Visceral extension	**0.024**	**0.020**
Vascular infiltration	**0.018**	0.538
Visceral infiltration	**0.004**	0.079
Morbidity	0.920	0.846
Severe morbidity (≥Clavien-Dindo 3a) [11]	0.822	0.764
Tumor size (<5 vs. 5–10 vs. >10 cm) *	**<0.001**	**<0.001**
Solitary vs. multifocal tumors	0.149	**0.001**
T stage	**<0.001**	**<0.001**
N stage	**<0.001**	**0.001**
M stage	**0.001**	**<0.001**
L stage	0.534	0.702
V stage	0.053	0.301
Pn stage	**0.003**	0.053
R stage	0.197	0.207
Grading	0.344	0.598
UICC stage	**<0.001**	**<0.001**

For survival analyses perioperative deaths (*n* = 18) were excluded; for multivariate analysis, *p*-values < 0.1 were further analyzed in Table 7 (parameters underlined); significant *p*-values <0.05 are bold; * size of the largest nodule obtained out of the histology report; UICC stage was not included in multivariate analysis; ASA = American Society of Anesthesiologists.

**Table 7:** Predictors of survival.

	Overall Survival			Recurrence-Free Survival		
	HR	95% CI	*p*-Value	HR	95% CI	*p*-Value
Gender				‡	‡	‡
Preoperative therapy				1.975	1.223–3.191	0.005
Major resection	‡	‡	‡	‡	‡	‡
Extended resection	‡	‡	‡	‡	‡	‡
Visceral extension	‡	‡	‡	‡	‡	‡
Vascular infiltration	‡	‡	‡			
Visceral infiltration	‡	‡	‡	‡	‡	‡
Tumor size (<5 vs. 5–10 vs. >10 cm) *	1.674	1.275–2.199	<0.001	1.503	1.166–1.936	0.002
Solitary vs. multifocal tumors				‡	‡	‡
T stage	1.333	1.134–1.566	<0.001	1.250	1.076–1.452	0.004
N stage	1.494	1.149–1.943	0.003	1.276	1.011–1.611	0.040
M stage	‡	‡	‡	2.812	1.507–5.247	0.001
V stage	‡	‡	‡			
Pn stage	‡	‡	‡	‡	‡	‡

For survival analyses perioperative deaths (*n* = 18) were excluded; for multivariate analysis, *p*-values <0.1 were further analyzed using backward selection (see Table 6); * size of the largest nodule obtained out of the histology report; ‡ eliminated value in backward selection; UICC stage was not included in multivariate analysis due to the fact that multiple tested parameters are included within the UICC staging.

**Table 8:** Review of the literature—survival after liver resection for iCCA.

Author	Reference	Year	Period of Data Collection (Years)	Number of Resections	Survival (%)			Median Survival (Months)
					1-Year	3-Years	5-Years	
Shimada	[21]	2007	7	74	69	35	31	24
Konstadoulakis	[22]	2008	15	54	80	49	25	22 *
Lang	[23]	2009	9.5	83	78	31	21	24
Jonas	[24]	2009	20	195	60.2	-	22.2	-
de Jong	[25]	2011	37	449	77.5	44.3	30.7	27.3
Farges	[26]	2011	11	116	92	69	45	58 *
Wang	[27]	2013	6	367	61.9	40.8	35.2	21
Bektas	[28]	2015	16	221 †	67	40	27	33
Bergeat	[29]	2015	16.7	107	79.8	49.4	34.6	32.8
Doussot	[30]	2015	20.3	188	91	59	45	48.7
Souche	[31]	2015	16	125	80	48	28	35
Buettner	[32]	2017	26.5	1057	78.9	51.4	39.2	37.4
Conci	[33]	2018	21	282	-	-	40.6	45.9
Bartsch	[14]	2018	8	102	72	32	21	21
Schnitzbauer	[34]	2020	10	488	-	-	-	32.2
Lang	pres.	2021	13	202	72	35	22	22.5

* R0 resections, † including explorations; pres. = present study.

## Data Availability

The datasets used and analyzed during the current study are available from the corresponding author on reasonable request.
